# Editorial: Trematode infection in ruminants, volume II

**DOI:** 10.3389/fvets.2023.1258751

**Published:** 2023-08-14

**Authors:** Hui Zhang, Jia Wang, Khalid Mehmood, Kun Li

**Affiliations:** ^1^College of Veterinary Medicine, South China Agricultural University, Guangzhou, Guangdong, China; ^2^College of Veterinary Medicine, Nanjing Agricultural University, Nanjing, China; ^3^Faculty of Veterinary and Animal Sciences, The Islamia University of Bahawalpur, Bahawalpur, Pakistan

**Keywords:** trematode infection, *Fasciola*, *Paramphistomum*, cattle, epidemiology

Fasciolosis is a zoonotic parasitic disease that is listed by the World Health Organization as one of the most neglected tropical diseases and has a dramatic impact on the health of humans and animals (1, 2). The intermediate host of *Fasciola* species is a freshwater snail (3, 4), form which the larvae of the parasite, called cercariae emerges in water and attach to aquatic plants to become encysted larvae called metacercariae. When metacercariae are ingested, they excyst in the gut and migrate within the liver of animals, while the adult flukes reside in the bile ducts of mammals such as sheep, goats, cattle, buffalo and humans (5–7). According to Mas-Coma et al. (5), the annual global economic loss due to fasciolosis is about US $3.2 billion because of reduced herd productivity, liver diseases, reduced carcass value and increased mortality. The high prevalence of fasciolosis, is one of the major threats to livestock in developing countries, with a negative impact on the production of ruminants (8, 9). *Fasciola hepatica* and *F. gigantica* greatly hinder the economic improvement of livestock production in many countries of the world, especially in humid tropical and subtropical countries (4, 10). The disease occurs mostly in grazing areas and marshlands, where livestock are infected through ingestion of grasses and other vegetations contaminated with metacercariae (4). *Fasciola* infections often reduce milk, meat and fur production by affecting the liver function of livestock, and lead to depression, weight loss, sterility and reduced immunity (3, 10). Although fasciolosis is a major constraint affecting the development of livestock production worldwide, but epidemiological data on ruminant fluke infection in many countries remains scarce.

*Paramphistomum* is a rumen parasite having a similar life cycle and infection route with *Fasciola*, and its infection is also common in ruminants, which is widely spread in tropical and subtropical areas of the world (11). Global climate change has led to the expansion of the distribution of those parasites, and the prevalence of them in non-endemic countries has greatly increased, which brings new challenges to disease prevention and control (12). Therefore, the aim of this study is to collate the research results in the fields related to those flukes' infections in order to improve the knowledge and understanding of those flukes' diseases, and to provide data support for the development of new strategies for the prevention and control of those flukes' diseases.

In this special e-collection there are 4 papers covering the above-mentioned aspects.

Américo et al. conducted a survey on the epidemiology of bovine fasciolosis in Brazil. They reported that Serrana Mesoregion of Santa Catarina, has been actually free for fasciolosis but some autochthonous cases of fasciolosis was detected in cattle in this region during 2018 to 2021. They collected the fecal samples from animals directly in eight municipalities and liver samples from three slaughter houses in which animals were brought from Serrana region for slaughtering purpose. The fecal samples were processed by sedimentation technique. Their results indicate that from 106 farms 9.2% (178/1,927) animals had fasciolosis but there is no heterogeneity between the municipalities, with the liver condemnation rate was 15.1% (1,744/11,556) in slaughtered cattle who never left the fasciolosis free Serrana area. Their study revealed the occurrence of autochthonous cases of bovine fasciolosis in the municipalities of the Serrana, and confirmed more positive animals.

Nyagura et al. presented a review report on the occurrence of fasciolosis in livestock, wildlife and humans and distribution of intermediate host in South Africa. They comprehensively reported the whole country scenario about fasciolosis which indicated that fasciolosis spread over 6 out of 9 provinces during 1960–2021. The diagnostic procedures in previous studies include morphological examination and identification of eggs and flukes while no molecular or confirmatory diagnosis of fasciolosis which may lead to false positive results. Furthermore, some studies reported the presence of *Fasciola* eggs and antibodies in humans, while prevalence of fasciolosis ranges 9.1–37.67% in livestock along with annual financial losses to livestock industry. Their review paper will contribute to overcome the lack of information on the prevalence of fasciolosis, significance of prospective research and significance of the disease on livestock community in South Africa.

Alvi et al. discussed about the prevalence and associated risk factors related to *Paramphistomum* in Pakistan which is a major threat to livestock industry. They conducted this research work on abattoir samples from rumen of small ruminants. They applied molecular method to get the prevalence of rumen fluke in small ruminants. Their results indicated that the prevalence in sheep was higher than goats although the difference was statistically not significant. The prevalence was higher in animals of <1 years, while male animals were more infected than female animals. The prevalence was less in grazing animals compared to stall fed animals and difference was statistically significant. The sequence analysis data of the *cox1* showed its origin from other countries including China, India, Laos, Saudi Arabia, and Thailand.

Meng et al. reported the serum cytokine patterns of primary and secondary *Fasciola*-infection in buffaloes. They divided the animals into three groups which were exposed to no infection, primary infection and secondary infection of *Fasciola gigantica*. Secondary infection was given 4 weeks after a primary infection to the secondary infection group only. The results indicated that there is no significant difference of serum cytokines in primary and secondary infected animals.

## Author contributions

HZ: Conceptualization, Funding acquisition, Visualization, Writing—review and editing, Investigation, Data curation, Formal analysis, Methodology, Project administration, Resources, Supervision, Validation. KM: Conceptualization, Writing—original draft, Writing—review and editing, Visualization, Data curation, Formal analysis, Investigation, Methodology, Project administration, Resources, Software, Supervision, Validation. JW: Writing—review and editing, Visualization, Data curation, Formal analysis, Investigation, Software, Supervision, Validation. KL: Writing—review and editing, Visualization, Data curation, Formal analysis, Investigation, Software, Supervision, Validation.
